# Associations Among CTA Collateral Scores, Multimodal MRI Lesion Volumes, and Clinical Severity in Acute Middle Cerebral Artery Infarction

**DOI:** 10.3390/jcm15062417

**Published:** 2026-03-21

**Authors:** Halil Gulluoglu, Hasan Armagan Uysal, Fatma Gulhan Sahbaz, Erkan Sahin

**Affiliations:** 1 Department of Neurology, Medicalpoint İzmir Hospital, Faculty of Medicine, Izmir University of Economics, 35330 İzmir, Türkiye; gul_shbz@hotmail.com; 2 Department of Neurology, Medicana International İzmir Hospital, Faculty of Medicine, Izmir University of Economics, 35330 İzmir, Türkiye; druysalarmagan@yahoo.com; 3 Department of Radiology, Medicalpoint İzmir Hospital, Faculty of Medicine, Izmir University of Economics, 35330 İzmir, Türkiye; erkan.sahin@mph.com.tr

**Keywords:** middle cerebral infarction, CTA, Souza CS system, MCA, PCA

## Abstract

**Background/objectives**: In this study, we aimed to investigate acute infarct volume on magnetic resonance diffusion-weighted imaging (MRI DWI), chronic infarct volume on FLAIR (fluid-attenuated inversion recovery), hypoperfused area volume on PWI (perfusion-weighted imaging), stenosis locations and rates on CT (computerized tomography) angiography, CT angiography collateral scoring, and correlation of background data and etiological factors with neurological clinical findings in patients with acute middle cerebral infarction. **Methods**: A total of 117 patients with MCA (middle cerebral artery) infarction were hospitalized for diagnosis and treatment after undergoing CT angiography within 9 h of symptom onset. Comparative results of Souza’s collateral score system, MRI parameters, and clinical outcomes were determined. **Results**: According to the Souza CS system, 23 patients were in the malignant profile and 94 in the good profile. There was a statistically significant difference between the malignant and benign profiles in terms of DWI volume, hypoperfused area volume on PWI sequence, white matter assessment using the Fazekas scale, and supratentorial and infratentorial chronic infarct volume on the FLAIR sequence (*p* < 0.001, *p* < 0.001, *p* < 0.001, *p* < 0.001, *p* < 0.001). **Conclusions**: Patients with a malignant profile on CTA may have a larger infarct volume and worse functional outcome. This should be recognized, and these patients should be followed up more carefully and attentively than those with good collateral scores.

## 1. Introduction

Stroke is one of the most important problems in the world in terms of serious disability and death at all ages. Large artery atherosclerosis, cardioembolism, and small vessel occlusion are the most important causes of acute ischemic stroke (AIS) [1,2]. The size of the ischemic lesion is determined by the specific artery involved, the location of the occlusion within that artery, and the adequacy of collateral circulation through arterial anastomoses [3]. Inflammation is the main mechanism by which cells in the penumbra degenerate, thereby influencing infarct size and early neurological deterioration [4,5]. Within the ischemic penumbra, inflammatory activation of microglia and endothelial cells leads to cytokine release, blood–brain barrier disruption, and leukocyte infiltration, which amplify oxidative stress and secondary neuronal injury. This process can convert potentially salvageable tissue into irreversible infarction, although later inflammatory responses also contribute to debris clearance and repair.

The development of collateral circulation plays a crucial role in the prognosis and treatment effectiveness following AIS. Without sufficient collateral flow, irreversible neuronal damage can occur within minutes [6].

After middle cerebral artery (MCA) occlusion, blood supply to the ischemic penumbra is provided by leptomeningeal or pial collaterals connecting to the distal branches of the anterior cerebral artery (ACA) and the posterior cerebral artery (PCA) and by blood supply to the distal segments of the MCA. The earlier and more extensive the development of collaterals, the better the neurological outcome and the smaller the infarcts [3,7].

Computed tomography angiography (CTA) has recently replaced magnetic resonance imaging angiography and catheter angiography because it can be performed safely and rapidly in the emergency department to evaluate collaterals [8,9]. Therefore, many CTA scores have been defined for assessing collateral status in AIS [10,11].

This study aimed to investigate the correlation between CTA collaterals and lesion volumes on simultaneous diffusion-weighted image (DWI), fluid attenuation inversion recovery (FLAIR), and perfusion-weighted image (PWI) sequences, as well as the Fazekas scale for white matter lesions [12]. Additionally, the study sought to determine the relationship between a malignant CTA collateral profile, DWI pattern, and neurological outcomes.

## 2. Materials and Methods

We conducted a retrospective analysis of patients with a confirmed diagnosis of AIS resulting from MCA occlusion and admitted to the Economy University Medical School Medicalpoint Hospital (İzmir, Türkiye) between 1 May 2021, and 1 September 2024.

A total of 117 patients with AIS due to MCA occlusion, who had undergone CTA within 9 h, were included in the study. Patients who had their first stroke, were older than 18 years, had MCA occlusion on CTA and received this diagnosis within the first 3 h, had a National Institutes of Health Stroke Scale (NIHSS) score between 5 and 24 at presentation, and had all DWI and FLAIR sequences obtained within 3 h were included in the study. Patients with transient ischemic attack, cerebral hemorrhage or hemorrhagic transformation, and patients with a history of another type of bleeding, infectious disease, demyelinating disease, rheumatic disease, vasculitis, malignancy, and chronic renal or hepatic insufficiency were excluded. Body mass indices (BMIs), comorbidities, and medications, including antiaggregant/anticoagulant drugs, were noted. We used the World Health Organization classification based on BMI [weight (kg)/height^2^ (m^2^)] to estimate body fat. According to this classification, BMI is considered as normal between 18.5 and 24.9, overweight between 25 and 29.9, grade 1 (moderate obesity) between 30 and 34.9, grade 2 (severe obesity) between 35 and 39.9, and grade 3 over 40 (very severe obesity) (13). Demographic data and hospitalization NIHSS scores (National Institutes of Health Stroke Scale) were recorded. NIHSS scores were classified as mild (0–5), moderate (5–15), severe (15–20), and very severe (20–42).

Briefly, all patients underwent an initial non-contrast CT examination followed by CT angiography (CTA) as part of the emergency stroke imaging protocol. CTA was performed within 9 h of symptom onset to assess vascular occlusion and collateral circulation. After the initial CT/CTA evaluation, multimodal magnetic resonance imaging was performed, including diffusion-weighted imaging (DWI), perfusion-weighted imaging (PWI), and FLAIR sequences. MRI examinations were obtained as soon as possible after admission, typically within approximately 3 h following CT/CTA, and always within the same 9 h diagnostic window from symptom onset. This standardized imaging workflow allowed for the assessment of both vascular status and early ischemic tissue changes in the acute phase of stroke.

**Figure 1:**
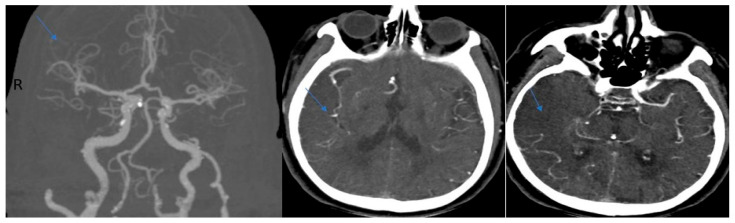
An 82-year-old woman with acute infarction of the right MCA. Atrial fibrillation was detected in the ECG. NIHHS:25, Souza’s CTA collateral score: 0. “Subtracted MIP images obtained from arterial phase images”. “Raw image in the axial plane obtained in the arterial phase after contrast injection’’. (“R” for right side of the image and Arrows show occluded arteries).

**Figure 2:**
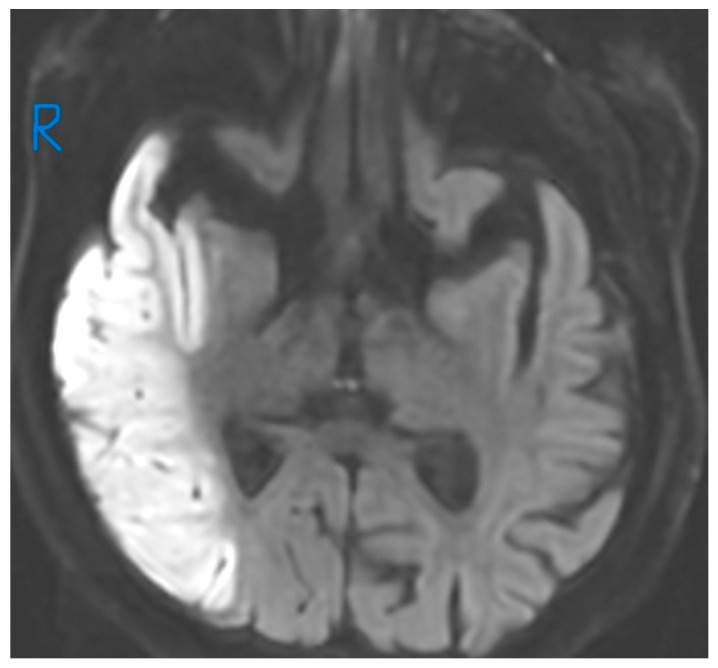
Neuroimaging findings taken 3 h after the onset of clinical signs. Acute infarct and its volume on DWI sequence—129,785 cm^3^. (“R” for right side of the image).

**Figure 3:**
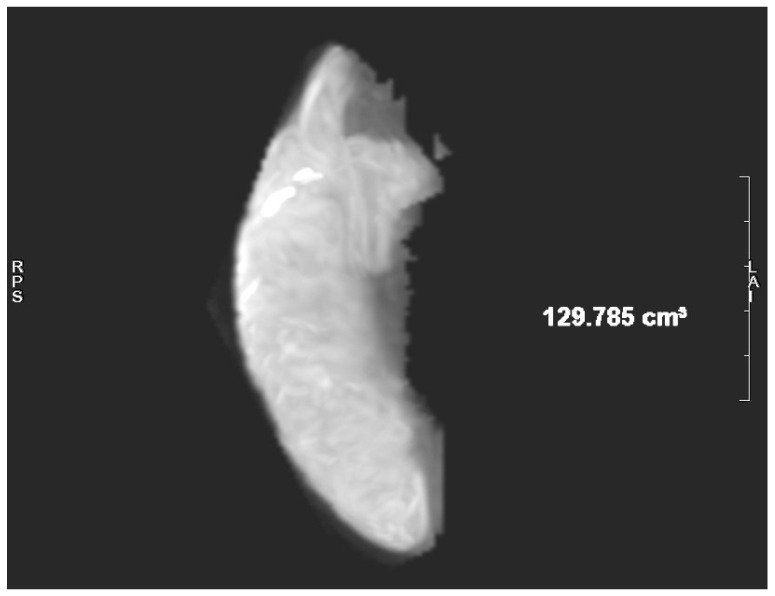
Acute infarction cite after 3 h and its volume on DWI sequence—129,785 cm^3^ (“RPS” right-posterior-superior, “LA” left-anterior).

**Figure 4:**
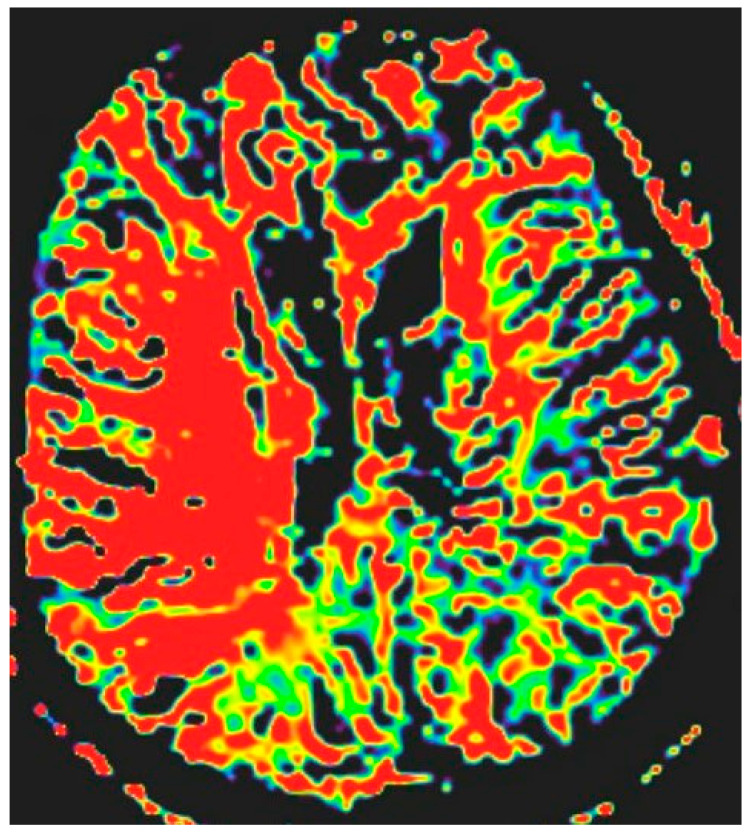
MTT sequence and its volume on PWI—281,345 cm^3^.

**Figure 5:**
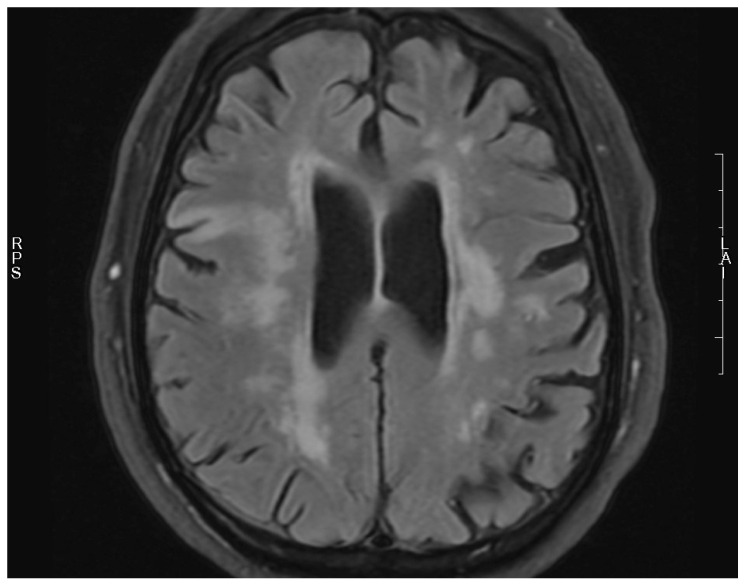
Chronic infarct and its volume on FLAIR sequence—106,593 cm^3^ (and stenosis location (internal carotid artery cavernous segment, “RPS” right-posterior-superior, “LA” left-anterior).

**Figure 6:**
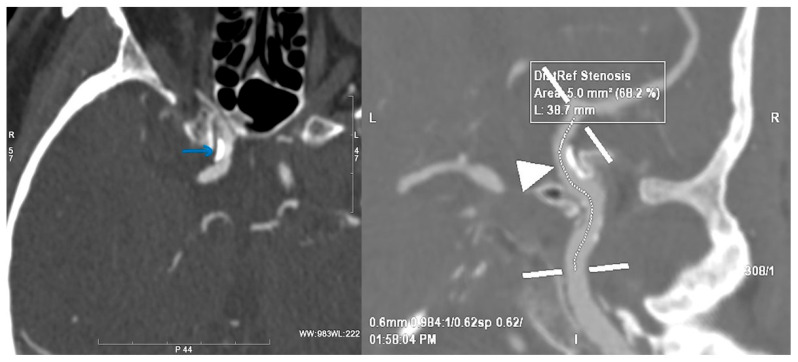
The ratio on CT angiography—62.8% (“R” for right side of the image, “L” for left side of the image).

Scanning protocols were performed on the GE Healthcare (2021, Milwaukee, IL, USA) 512-slice CT scanner. All patients included in the study initially underwent standard non-contrast CT. A total of 50 mL of contrast material (iohexol, Omnipaque, and 300 mg iodine/mL) was used at 5 mL/s for CTA extraction. Multiplane 7 mm maximum intensity projection (MIP) reconstructions and 4 mm axial reformats, or CTA source images, were acquired. General Electric Health Care (United States, 2021) 3 Tesla magnetic resonance imaging sequences were performed. DWI-PWI and FLAIR sequences were obtained within the first 9 h at the time of diagnosis. An open-source OsiriX DICOM imager (version 12.5.2) was used for quantitative volumetric analysis of infarcts. The closed polygon tool was used to manually delineate infarct areas, creating a region of interest for each sequence. CTA, DWI, FLAIR, and PWI sequences were evaluated independently by a neurologist (HG) and a neuroradiologist (ES). The Souza collateral scoring methodology [13] is thoroughly described by Souza et al. and has demonstrated high inter-observer concordance. Pre-contrast CT head scans were independently reviewed by two neuroradiologists (with 5 years of stroke imaging experience) who were unaware of the patient’s clinical symptoms. Reviewers were blinded to patients’ demographic data, clinic, imaging follow-up, and treatment. Carotid CT angiography was performed with a General Electric (GE) Revolution 512-slice computed tomography device (General Electric Revolution, 2021, Milwaukee, IL, USA). The examination was performed using a Medrad Certegra (Bayer, 2021, Berlin, Germany) automatic injector. A total of 50 cc of 350 mgI/mL Iohexol (Copaq, Koçsel İlaç San. ve Tic. A.Ş., Istanbul, Türkiye) was administered through the antecubital vein, followed by isotonic 0.9% NaCl (40 cc) at a rate of 4 mL/sec with a slice thickness of 0.625 mm. The images obtained were evaluated on the GE AW server Workstation in axial, sagittal, and coronal planes using the volume rendering protocol (VRT) and the maximum intensity protocol (MIP). An MRI perfusion examination was obtained with the GE 3 Tesla Premier (GE, 2021, Milwaukee, IL, USA) with a 21-channel head and neck coil. During examination, Gadobutrol 0.1 mmol/kg (Gadovist 1 mmol/mL, Bayer Healthcare Pharmaceuticals Inc., Berlin, Germany) was used at a rate of 4 mL/sec through the antecubital vein with a Medrad automatic injector (Bayer, Berlin, Germany). Cerebral blood flow (CBF), cerebral blood volume (CBV), and mean transit time (MTT) maps were generated with a single-shot gradient-echo echoplanar sequence using the dynamic susceptibility contrast MRI (DSC MRI) technique. To evaluate the CTA collateral status, the Souza collateral scoring system was used [13]: 0 = absent collaterals >50% M2 territory, 1 = diminished collaterals >50% M2 territory; 2 = diminished collaterals, <50% M2 territory; 3 = collaterals equal to contralateral side; and 4 = increased collaterals. Patients were dichotomized into 2 categories: CS = 0 (malignant profile) or CS > 0 (good profile) (Figure 1, Figure 2, Figure 3, Figure 4, Figure 5 and Figure 6).

SPSS 25.0 (IBM Corp., Armonk, NY, USA) and MedCalc 14 (Acacialaan 22, B-8400 Ostend, Belgium) programs were used to analyze the variables. The normality of the data was evaluated using the Shapiro–Wilk test, and homogeneity of variance was evaluated using the Levene test. The Mann–Whitney U Monte Carlo simulation results were used to compare two groups according to quantitative variables. In the comparison of categorical variables, the Pearson chi-square test, Fisher’s exact test, and Fisher–Freeman–Halton test were tested with the Monte Carlo simulation technique. Since there were too many significant variables, variable selection was performed using the backward method in logistic regression. Sensitivity, specificity, positive predictive ratio (positive predictive and positive predictivity), and negative predictive ratio (negative predictive and negative predictivity) were examined and expressed by ROC (receiver operating curve) curve analysis for the relationship between the classification separated by the cut-off value calculated according to the variables of the selected numerical variable groups and the actual classification. Finally, a logistic regression test utilizing the backward method was used to determine the cause-and-effect relationship between the categorical dependent variable and the explanatory variables. Odds ratios (ORs) with corresponding 95% confidence intervals (CIs) were calculated to estimate the strength of association between these variables and malignant collateral status. Quantitative variables were expressed as mean (standard deviation) and median (minimum–maximum) in the tables, while categorical variables were expressed as n (%). Variables were analyzed at the 95% confidence level, and *p*-values < 0.05 were considered statistically significant.

## 3. Results

### 3.1. Sociodemographic and Clinical Characteristics

A total of 69 patients were male (58.9%), and 48 were female (41.1%), with an age range of 54–85 years (mean = 71). The mean time to the emergency department was 4.5 (SD = 1.55) hours, the mean CTA time was 5 (SD = 1.49) hours, and the mean NIHSS score on arrival was 15.3 (SD = 9.9). The cause of stroke remained undetermined in 30 patients. Among the remaining cases, 39 were attributed to large artery atherosclerosis (thrombus/embolism), 31 to cardioembolism (high or intermediate risk), and 17 to small-artery occlusion (lacunar infarction). The BMI ranged from 23.9 to 39.9 (mean = 33.65 kg/m^2^), and only seven patients had a normal body weight. The remaining 28 patients were pre-obese; 40 were grade 1 obese, and 42 were grade 2 obese. The average pulse rates of the patients upon arrival at the emergency room ranged from 65/min to 148/min (mean = 111/min). While the mean systolic blood pressures were 162.5 mmHg, the mean diastolic blood pressures were 90.25 mmHg. When the patients presented to the emergency department, the minimum blood glucose level was 109 mg/dL, and the maximum blood glucose level was 276 mg/dL (mean = 159 mg/dL).

### 3.2. Comorbidities

Hypertension was present in 106 patients (90.6%), with a duration ranging from three to 36 years (mean = 13 years). A total of 62 patients (53%) had diabetes mellitus, with a minimum of four years and a maximum of 21 years (mean = 11 years).

Coronary artery disease was present in 72 patients (61.5%) for a minimum of three years and a maximum of 17 years (mean = 8.5 years). Seventy-two patients were hyperlipidemic, and hyperlipidemia was present for a minimum of five years and a maximum of 23 years (mean = 10.5 years). Thirty-one of the patients (26.4%) had a history of cardiac arrhythmia. There was a history of antiaggregant use in 78 patients (66.6%) and of anticoagulant use in 31 patients (26.5%). Fifty-four patients (46.2%) had used alcohol for a minimum of 10 years and a maximum of 45 years (mean = 26 years). Seventy-five patients (64.1%) had a history of smoking, and the duration of smoking was a minimum of 10 years and a maximum of 45 years (mean = 28 years; Table 1).

The pulse rate was 79/min (65–93/min) in patients with a malignant profile and 115/min (65–148/min) in those with a good profile (*p* < 0.001). The mean duration of coronary artery disease was 14 years (9–17 years) under a malignant profile and eight years (3–14 years) under a good profile (*p* < 0.001). Patients with a malignant profile had a mean hyperlipidemic course of 13 years (10–16 years), while patients with a benign profile were hyperlipidemic for nine years (5–23 years; *p* = 0.001). Specifically, cardiac arrhythmia and anticoagulant use were observed exclusively in patients with a good collateral profile, whereas no patients with a malignant collateral profile had a history of arrhythmia or anticoagulant therapy (*p* = 0.001; Table 1).

### 3.3. Clinical Characteristics

In the Souza CS assessment, 23 patients were classified with a CS score of 0 (19.7%), 20 with a CS score of 1 (17.1%), 12 with a CS score of 2 (10.3%), 41 with a CS score of 3 (35%), and 21 with a CS score of 4 (17.9%). According to this system, 23 patients (19.7%) were assigned as malignant profile, while 94 patients (80.3%) were assigned as benign profile. The Souza CS system and clinical disability comparison results showed that the mean NIHSS value for patients with a malignant profile was 28 (25–30), while the mean NIHSS value for patients with a benign profile was 8 (3–31; *p* < 0.001).

Comparison results of the Souza CS system and MRI parameters analysis revealed distinctions. The count of acute infarcts on DWI was one (1/1) in the malignant profile and one (1/4) in the benign profile, indicating a statistically significant difference (*p* = 0.005). The mean DWI volume in patients with a malignant profile was 296.85 (259.95/312.42) cm^3^, while this was significantly lower in patients with a benign profile [26.39 (2.88/298.53) cm^3^], revealing a statistically significant difference (*p* < 0.001). When we compared the volume of the hypoperfused area in the PWI sequence, it was found to be 299.82 cm^3^ (266.95/319.99) in the malignant profile and 34.23 cm^3^ (2.88/322.21) in the benign profile, revealing a statistically significant difference (*p* < 0.001). In the white matter evaluation using the Fazekas scale, grade 3 lesions were detected in all patients with a malignant profile, while 11 (9.4%) patients with a benign profile had grade 1 lesions, 26 (22.2%) patients had grade 2 lesions, and 80 (68.4%) patients had grade 3 lesions. Statistical significance was found in the statistical comparison (*p* < 0.001). In the FLAIR sequence for MRI, the number of chronic infarcts in the supratentorial region was 18 (9/24) in the benign profile and 13.5 (3/26) in the malignant profile, revealing a statistically significant difference (*p* = 0.003). The mean supratentorial chronic infarct volume on the FLAIR sequence in patients with a malignant profile was 18.71 cm^3^ (11.54/30.54), whereas it was 14.94 cm^3^ (5.83/38.67) in patients with a benign profile; the difference was statistically significant (*p* = 0.002). Looking at the infratentorial region, the mean volume of the chronic infarct in the FLAIR sequence was 2.83 cm^3^ (1.86/9.51) in the malignant group and 2.14 cm^3^ (0/5.48) in the benign group, again revealing a statistical significance (*p* = 0.004). In the FLAIR sequence study of the infratentorial region, the number of chronic infarcts was four (1/6) in the benign profile and three (0/7) in the malignant profile (Table 2 and Table 3). Accuracy rates after comparison of MRI and CTA parameters with collateral scoring system in Souza CTA are depicted in full detail in Table 3.

Atherosclerotic plaque volume in the arcus aorta on the CTA was 2.3 cm^3^ (1.32/2.99) in the malignant profile and 1.96 cm^3^ (0/5.69) in the benign profile (*p* = 0.151). When the length of atherosclerotic plaque in the arcus aorta was evaluated by CTA, it was 19.82 mm (4.15/25.75) in the malignant group and 14.39 mm (0/31.81) in the benign group (*p* = 0.05). Stenosis rates in the right common carotid artery (CCA), right internal carotid artery (ICA), M1 branch of the MCA, right vertebral artery (VA), basilar artery (BA), P2 segment of the right posterior cerebral artery (PCA), and P2 segment of the left PCA were compared in the malignant and benign groups on CTA. Accordingly, there was a statistical difference between the malignant and benign profiles in the right CCA and the M1 segment of the MCA on both the affected (100% vs. 0) and unaffected sides (21% vs. 0; *p* < 0.001). In the ICA (0 vs. 31.35%), a statistical difference was detected only on the affected side (*p* < 0.001). Stenosis rates on the right VA on CTA (0 vs. 10.85%) did not differ on the affected site (*p* = 0.244). In the right VA, a statistical difference was observed on the unaffected side (*p* = 0.012). While there was a statistical difference between malignant and benign groups in the right P2 segment of PCA (12.3%; *p* = 0.018), there was no statistical difference in the basilar artery and left P2 segment of PA (*p* = 0.214, *p* = 0.960; Table 2 and Table 3).

A multivariable logistic regression analysis was performed to identify independent predictors of a malignant collateral profile. Variables with strong diagnostic performance in the ROC analysis and clinical relevance were included in the model. Higher NIHSS score (≥24), larger infarct volume on DWI (≥259.9 mm^3^), and higher stenosis rate in the M1 segment of the MCA were independently associated with malignant collateral status. In addition, the presence of stenosis in the right common carotid artery was also significantly associated with a malignant collateral profile. Among these predictors, DWI infarct volume showed the strongest association with malignant collateral status (OR = 12.4, 95% CI = 3.1–49.7, *p* < 0.001).

## 4. Discussion

This study investigated the relationship between CTA-based collateral circulation status and clinical, as well as radiological, parameters in patients with acute middle cerebral artery infarction. Overall, our findings demonstrate that patients with a malignant collateral profile exhibit a substantially greater ischemic burden and more severe neurological impairment compared with those with a benign collateral profile. Specifically, malignant collateral status was associated with significantly larger acute infarct volumes on diffusion-weighted imaging, greater hypoperfused areas on perfusion-weighted imaging, and markedly higher NIHSS scores at presentation. In addition, several vascular parameters assessed by CT angiography—particularly stenosis in the M1 segment of the middle cerebral artery and the right common carotid artery—were significantly associated with malignant collateral status. Multivariable analysis further identified larger DWI infarct volume, higher NIHSS score, and MCA-M1 stenosis as independent predictors of a malignant collateral profile. Taken together, these results highlight the close relationship between impaired collateral circulation, increased infarct burden, and greater clinical severity in acute ischemic stroke, emphasizing the potential value of early CTA-based collateral assessment in prognostic stratification and treatment planning.

Cerebral infarction rates following acute MCA stroke depend on the extent of collateral circulation and the ability of tissue cells to withstand ischemic hypoxia. Therefore, even with good collateral circulation, in cases with poor tolerance to ischemia and hypoxia, ischemia can lead to rapid cerebral infarction, a large infarct area, and a poor prognosis [14]. Although patient-specific factors influence this condition, studies have shown that intracerebral collateral circulation also plays a significant role. The traditional angiographic grading system has demonstrated a significant relationship between collateralization and positive clinical outcomes [15,16,17,18]. In this study, patients with a malignant CTA Souza score profile had larger infarct volumes and poorer functional clinical outcomes.

Digital subtraction angiography (DSA, considered the standard but invasive technique) [19,20], CTA [21], computed tomography perfusion [22], and magnetic resonance angiography [23] are imaging modalities used to assess collateral flow. Among these examinations, CTA is ahead of the others because of its easy access, ease of use, non-invasiveness, and rapid results [10,11,24,25]. Despite the magnitude of scores that have been used for CTA, such as Souza collateral score [13], Maas collateral score [25], Alberta Stroke Programme Early CT Score (ASPECTS) [26], Christoforidis collateral score [27], Miteff collateral score [7], Tan collateral score [28], and Careggi collateral score [29], there is still no consensus on this issue.

This study also found that despite the simple binary categorization, the presence of collaterals was the only radiological predictor of a positive outcome [16]. Since infarct expansion is influenced by collateral status, collateral status is an important predictor of clinical outcome in many studies, independent of successful recanalization [30,31]. The status of collateral flow is strongly and independently associated with clinical outcomes in acute ischemic stroke [30,31,32]. Multiphase CTA consists of non-contrast (NCCT), arterial (CTA-AP), and delayed (CTA-DP) phases and is useful for assessing collateral status and selecting patients for endovascular treatment [32,33]. In this study, we assumed that the Souza collateral score on multiphase CTA reflects both the final infarct volume and the clinical outcome.

In our study, we used the Souza CS system because it is simple, reliable, easy to apply, provides a faster real-time assessment, and is more suitable for patient selection for intra-arterial therapy when needed. Patients with a malignant profile have a risk of having a large infarct volume before treatment. Therefore, they are less likely to benefit from revascularization treatment. Based on this prediction, patients can be identified and treatment decisions made. Puetz et al. [34] performed CTA on stroke patients with a malignant profile. The authors reported that of 114 anterior circulation stroke patients treated with IV tPA within three hours and evaluated according to the 20-point ASPECT score, the score was 10 (malignant profile) in 24 (21%) cases, and half of these patients died.

The Fazekas scale is used to quantify the amount of white matter T2 hyperintense lesions, usually attributed to chronic small vessel ischemia. This classification was proposed by Fazekas et al. [12] in 1987 and is the most commonly used scale to describe the severity of white matter disease. Clinical use is limited because not all lesions are caused by small vessel disease. In the Fazekas scale, white matter is divided into periventricular and deep white matter, and grading is performed depending on the size and combination of lesions in the region.

Cerebral infarction rates after acute SVO depend on collateral circulation compensation and the ability of brain tissue cells to tolerate ischemic hypoxia, as cells in different individuals exhibit varying tolerances to ischemia and hypoxia. Therefore, even with good collateral circulation, in cases with poor tolerance to ischemia and hypoxia, ischemia can lead to rapid cerebral infarction, a large infarct area, and poor prognosis. [14]. There was an evident difference between malignant and good profiles in terms of DWI volume, hypoperfused area volume on PWI sequence, white matter assessment using the Fazekas scale, and supratentorial and infratentorial chronic infarct volume on the FLAIR sequence. We also found a difference between the malignant and benign profiles in the M1 segment of the right CCA and the MCA (both right and left, both affected and unaffected sides), ICA (only the affected side), right VA (only the unaffected side), and right P2 segment of the PCA. In addition to the final infarct volume, the CTA collateral score also helps us predict patients’ functional outcome after ischemia. Patients classified with a collateral score indicative of a malignant profile exhibit larger infarct volumes and experience poorer functional outcomes. Bang et al. [35] showed that patients with a good angiographic profile had higher intra-arterial treatment response rates and recanalization rates and lower increases in infarct volume. Angermaier et al. [36] reported that the CTA collateral score was an independent determinant of the final infarct volume in infarct patients undergoing endovascular treatment. In a study by Rosenthal et al. [37], it was emphasized that the positive effect of CTA collaterals was greater in incompletely recanalized patients than in fully recanalized patients. Lima et al. [24] also emphasized that the beneficial effects of collaterals in untreated patients should be known. Nevertheless, further studies are needed to determine the effects of CTA collateral scores with good and malignant profiles on infarct volumes and clinical outcomes.

Contemporary studies also support the vital role of collateral circulation, as evaluated by CTA, in determining infarct progression and functional outcomes in acute ischemic stroke. Studies including ASPECTS-based collateral examinations showed that poor collateral status was associated with rapid infarct growth and adverse neurological outcomes, underscoring the importance of early collateral evaluation in decision-making [38]. Additionally, Elijovich et al. stated that CTA-based collateral grading reliably indicated tissue viability and long-term functional prognosis, supporting the value of collateral imaging as a surrogate marker of penumbral preservation in acute MCA occlusion [39]. Furthermore, it has been suggested that modern multiphase CTA collateral mapping was closely associated with perfusion imaging and might improve baseline lesion assessment and prognostic stratification in acute anterior circulation stroke beyond traditional single-phase scores [40]. These results are consistent with our findings, underscoring the prognostic importance of CTA collateral profiles in predicting infarct burden and clinical outcomes.

Our study has some limitations. In particular, as a single-center study and given the lack of other stroke centers in the region, it is challenging to compare our patients with those in similar cohort studies. Additionally, due to imaging limitations and the lack of continuous arterial monitoring, the precise timing of recanalization within 24 h of presentation cannot be determined. Although our patient cohort was smaller than in other series, we still had sufficient patients to perform progression analysis.

## 5. Conclusions

In conclusion, CTA-based collateral circulation status is strongly associated with infarct burden and neurological severity in patients with acute middle cerebral artery infarction. Patients with a malignant collateral profile demonstrated significantly larger infarct volumes on diffusion-weighted imaging, greater hypoperfused areas on perfusion-weighted imaging, and higher NIHSS scores at presentation. Additionally, vascular abnormalities detected on CT angiography—particularly stenosis of the MCA M1 segment and the right common carotid artery—were significantly related to malignant collateral status, with larger DWI infarct volume emerging as the strongest independent predictor. These findings highlight the clinical value of early collateral assessment using CTA as a rapid imaging marker for identifying patients at risk of extensive ischemic injury and severe neurological deficits. Integrating collateral evaluation into the initial imaging workflow may therefore improve prognostic assessment and assist in guiding therapeutic decision-making in acute ischemic stroke.

## Figures and Tables

**Table 1:** Comparative analysis of malignant and non-malignant collateral score groups.

	CTA	CS	
	<1	≥1	
	(*n* = 23)	(*n* = 94)	*p*
Age, median (min/max)	75 (60/82)	70.5 (54/85)	**0.019 ^u^**
Gender (male), *n* (%)	15 (65.2)	54 (57.4)	0.637 ^c^
Mean time of admission to the emergency department, median (min/max) (hours)	2.1 (0.5/3)	2.5 (0.6/3)	**0.022 ^u^**
Causes of stroke, *n* (%)			
Large artery atherosclerosis (embolus/thrombosis)	23 (100)	16 (17)	
Cardioembolism (high risk/medium risk)	0 (0)	31 (31.8)	
Small vessel occlusion (lacune)	0 (0)	17 (17.6)	
Stroke of undetermined etiology	0 (0)	30 (32.9)	
BMI, median (min/max)	29.9 (23.9/37.6)	34.1 (23.9/39.9)	**0.008 ^u^**
**BMI, *n* (%)**			
Normal	3 (13)	4 (4.7)	
Pre-obesity	10 (43.5)	17 (20)	
Grade 1 obese	7 (30.4)	30 (35.3)	
Grade 2 obese	3 (13)	34 (40)	
NIHSS score, median (min/max)	28 (25/30)	8 (3/31)	**<0.001 ^u^**
Pulse frequency rates of the patients when they came to the emergency room, minutes, median (min/max)	79 (65/93)	115 (65/148)	**<0.001 ^u^**
Mean systolic blood pressures, mmHg, median (min/max)	165 (123/177)	159 (125/204)	0.631 ^u^
Mean diastolic blood pressures, mmHg, median (min/max)	91 (79/105)	89.5 (69/121)	0.085 ^u^
Blood glucose of the patients when they came to the emergency room, mg/dL, median (min/max)	159 (113/196)	159 (109/276)	0.503 ^u^
Hypertension, *n* (%)	23 (100)	83 (88.3)	0.118 ^f^
Duration of hypertension, years, median (min/max)	13 (5/36)	12 (3/35)	0.061 ^u^
Duration of diabetes mellitus, years, median (min/max)	13 (7/19)	11 (4/21)	0.440 ^u^
Coronary artery disease, *n* (%)	16 (69.6)	56 (59.6)	0.476 ^c^
Duration of coronary artery disease, years, median (min/max)	13.5 (9/17)	8 (3/14)	**<0.001 ^u^**
Hyperlipidemia, *n* (%)	16 (69.6)	56 (59.6)	0.476 ^c^
Duration of hyperlipidemia, years, median (min/max)	12.5 (10/16)	9 (5/23)	**0.001 ^u^**
History of antiaggregant use, *n* (%)	16 (69.6)	62 (66)	0.810 ^f^
History of alcohol use, *n* (%)	15 (65.2)	37 (39.4)	**0.035 ^u^**
Duration of alcohol use, years, median (min/max)	28 (18/41)	26 (10/45)	0.744 ^u^
History of smoking, *n* (%)	17 (73.9)	58 (61.7)	0.337 ^f^
Duration of smoking, years, median (min/max)	29 (20/45)	28 (10/45)	0.342 ^u^

^f^ Fisher exact test (Monte Carlo), ^c^ Pearson chi-square test (Monte Carlo), ^u^ Mann–Whitney U test (Monte Carlo); CTA < 1 corresponds to CS = 0.

**Table 2:** Comparison of MRI and CTA parameters’ collateral score system on Souza CTA.

	CTA	CS	
	<1	≥1	
	(*n* = 23)	(*n* = 94)	*p*
Number of acute infarcts on DWI, median (min/max)	1 (1/1)	1 (1/4)	**0.005 ^u^**
Volume in DWI, cm^3^, median (min/max)	296.85 (259.95/312.42)	26.39 (2.88/298.53)	**<0.001 ^u^**
Volume of hypoperfused area in PWI sequence, cm^3^, median (min/max)	299.82 (266.95/319.99)	34.23 (2.88/322.21)	**<0.001 ^u^**
White matter evaluation using the Fazekas scale, median (min/max)	3 (3/3)	3 (1/3)	**<0.001 ^u^**
Number of chronic infarcts for supratentorial region on the FLAIR MRI sequence, median (min/max)	18 (9/24)	13.5 (3/26)	**0.003 ^u^**
Volume of chronic infarct for supratentorial region on the FLAIR sequence, cm^3^, median (min/max)	18.71 (11.54/30.54)	14.94 (5.83/38.67)	**0.002 ^u^**
Number of chronic infarcts for the infratentorial region on the FLAIR MRI sequence, median (min/max)	4 (1/6)	3 (0/7)	**0.004 ^u^**
Volume of chronic infarct in the FLAIR sequence for the infratentorial region, cm^3^, median (min/max)	2.83 (1.86/9.51)	2.14 (0/5.48)	**0.004 ^u^**
Atherosclerotic plaque volume in the arcus aorta on CTA, cm^3^, median (min/max)	2.3 (1.32/2.99)	1.96 (0/5.69)	0.151 ^u^
Length of atherosclerotic plaque in the arcus aorta on CTA, mm, median (min/max)	19.82 (4.15/25.75)	14.39 (0/31.81)	0.050 ^u^
Stenosis rates in the right ICA on CTA (unaffected site, left for control) (unaffected site, right for control), median (min/max), %	17.3 (10.9/25.4)	17.35 (0/47.6)	0.974 ^u^
Stenosis rates in the right ICA on CTA (unaffected site, left for control) (unaffected site, left for control), median (min/max), %	17.3 (10.9/25.4)	17.35 (0/47.6)	0.974 ^u^
Stenosis rates in the right VA on CTA, (unaffected site, right for control), median (min/max), %	14.2 (0/25.2)	4.8 (0/25.3)	**0.012 ^u^**
Stenosis rates in the VA on CTA, (unaffected site, left for control), median right (min/max), %	14.2 (0/25.2)	4.8 (0/25.3)	**0.012 ^u^**
Stenosis rates in the BA on CTA, (median (min/max), %	19.5 (0/29.6)	13.8 (0/45.7)	0.214 ^u^

^u^ Mann–Whitney U test (Monte Carlo); min: minimum; max: maximum.

**Table 3:** Accuracy rates after comparison of MRI and CTA parameters with collateral scoring system in Souza CTA.

Reference: ≥1	Cut-Off	Sensitivity	Specificity	+PV	−PV	OR 95% CI *p*	AUC ± SE.	*p*
Age	≤65	≤66	≤67	≤68	≤69		≤70	≤71
Mean time of admission to the emergency department	>4.5	41.49	86.96	96.9	26.7		0.652 ± 0.060	**0.011**
NIHSS score	≤24	84.04	100.00	100.00	60.5	8.6 2.3–31.9 <0.001	0.910 ± 0.027	**<0.001**
Number of acute infarcts on DWI	>1	28.72	100.00	100.00	25.6		0.644 ± 0.055	**0.001**
Volume in DWI	≤259.89	86.17	100.00	100.00	62.2	12.4 3.1–49.7 <0.001	0.951 ± 0.018	**<0.001**
Fazekas scale for white matter lesions	≤2	39.36	100.00	100.00	28.7		0.697 ± 0.050	**<0.001**
Number of chronic infarcts for supratentorial region on the FLAIR MRI sequence	≤12	44.68	95.65	97.7	29.7		0.696 ± 0.052	**<0.001**
Volume of chronic infarct in the FLAIR sequence for infratentorial region	≤1.68	41.49	100.00	100.00	29.5		0.696 ± 0.055	**<0.001**
Stenosis rates in the right CCA on CTA (unaffected site, right for control)	≤0	67.02	100.00	100.00	42.6	3.2 1.1–9.5 0.031	0.848 ± 0.035	**<0.001**
Stenosis rates in the M1 branch of MCA on CTA (affected site, right for control)	≤32.6	78.72	100.00	100.00	53.5	5.7 1.7–18.8 0.004	0.894 ± 0.029	**<0.001**
Stenosis rates in the M1 branch of MCA on CTA, (unaffected site, right for control)	>0	64.89	100.00	100.00	41.1		0.824 ± 0.037	**<0.001**
Stenosis rates in the right VA on CTA, (affected site, right for control)	>0	54.26	65.22	86.4	25.9		0.573 ± 0.068	0.286
Stenosis rates in the right P2 segment of PCA on CTA	≤15.8	86.17	0.00	77.9	0.0		0.503 ± 0.064	0.965

AUC: area under the ROC curve; SE: standard error; +PV: positive predictive value; −PV: negative predictive value.

## Data Availability

Data supporting reported results can be obtained from the corresponding author upon reasonable request.

## Abbreviations

The following abbreviations are used in this manuscript:

AIS	Acute ischemic stroke
BA	Basilar artery
BMI	Body mass index
CBF	Cerebral blood flow
CBV	Cerebral blood volume
CCA	Common carotid artery
CS	Collateral score
CTA	Computed tomography angiography
DWI	Diffusion-weighted imaging
DSA	Digital subtraction angiography
FLAIR	Fluid-attenuated inversion recovery
ICA	Internal carotid artery
MCA	Middle cerebral artery
MIP	Maximum intensity projection
MRI	Magnetic resonance imaging
MTT	Mean transit time
NCCT	Non-contrast computed tomography
NIHSS	National Institutes of Health Stroke Scale
PCA	Posterior cerebral artery
PWI	Perfusion-weighted imaging
ROI	Region of interest
VA	Vertebral artery
